# First detection of the S989P+V1016G+D1763Y haplotype and expansion of voltage-gated sodium channel mutations in *Aedes aegypti* in Taiwan in 2016–2023

**DOI:** 10.1371/journal.pntd.0012768

**Published:** 2025-01-06

**Authors:** Han-Hsuan Chung, Hwa-Jen Teng, Chin-Gi Huang, I-Jung Tsai, Hung-Jui Chen, Osamu Komagata, Shinji Kasai, Kun-Hsien Tsai, Shiu-Ling Chen

**Affiliations:** 1 Center for Diagnostics and Vaccine Development, Centers for Disease Control, Ministry of Health and Welfare, Taipei, Taiwan; 2 Institute of Environmental and Occupational Health Sciences, College of Public Health, National Taiwan University, Taipei, Taiwan; 3 National Mosquito-Borne Diseases Control Research Center, National Health Research Institutes, Miaoli, Taiwan; 4 Department of Medical Entomology, National Institute of Infectious Diseases, Shinjuku-ku, Tokyo, Japan; 5 Department of Public Health, College of Public Health, National Taiwan University, Taipei, Taiwan; Kenya Agricultural and Livestock Research Organization, KENYA

## Abstract

**Background:**

*Aedes aegypti* transmits various arthropod-borne diseases such as dengue, posing a significant burden to public health in tropical and subtropical regions. Pyrethroid-based control strategies are effective in managing this vector; however, the development of insecticide resistance has hindered these efforts. Hence, long-term monitoring of insecticide resistance in mosquito populations is crucial for effective vector and disease control.

**Methodology/principal findings:**

In this study, we identified insecticide resistance due to a voltage-gated sodium channel (*vgsc*) mutation in *Ae*. *aegypti* in Taiwan between 2016 and 2023. In total, 1,761 field-caught *Ae*. *aegypti* samples from Tainan, Kaohsiung, and Pingtung were genotyped. The frequencies of S989P, V1016G, T1520I, F1534C, and D1763Y amino acid variants increased over the surveillance period. A T1520I mutation was detected for the first time and has since rapidly spread throughout Taiwan. The triple-mutant haplotype PGTFY was first documented in *Ae*. *aegypti*. Moreover, the unmutated haplotype vanished in Taiwan, suggesting that the *vgsc* mutations were fixed in local populations of *Ae*. *aegypti*. Five resistance-associated genotypes, SVTCD/SVTCD, SGTFY/PGTFD, SVTCD/SGTFY, PGTFD/PGTFD, and SVTCD/PGTFD, exhibited an increased frequency and accounted for 76% of the total field population. We also detected the resistant genotype SVICD/PGTFD, and its frequency increased 13-fold in the field between 2016 and 2023. Moreover, we also observed that mutations differed geographically, with S989P mainly found in Kaohsiung and V1016G in Kaohsiung and Pingtung. The frequency of T1520I was noticeably higher in Kaohsiung, and D1763Y occurred mainly in Tainan.

**Conclusions/significance:**

The emergence and expansion of mutations along with the disappearance of wild-type mosquitoes in Taiwan underscores the threat of resistance and difficulty of mosquito control in Taiwan as well as globally. This study determined the insecticide resistance status of *Ae*. *aegypti* in Taiwan, and the findings will be helpful for resistance monitoring in areas where pyrethroids are used to control *Ae*. *aegypti*.

## Author summary

We conducted an eight-year insecticide resistance surveillance study on *vgsc* mutations to monitor resistance trends and understand the role of *vgsc* mutations in *Aedes aegypti* in Taiwan. Mutations S989P, V1016G, T1520I (first documented in Taiwan), F1534C, and D1763Y were detected, and their frequencies increased over time. We observed that *Ae*. *aegypti* with resistance-associated *vgsc* genotypes comprised the majority of the field population in later years of surveillance, and the unmutated haplotype disappeared; all surveyed field *Ae*. *aegypti* harbored at least one *vgsc* mutation. Three haplotypes were first detected in Taiwan: PGTFY, PGTCD, and SVICD. This study presents the first report of PGTFY in *Ae*. *aegypti*, and determining its role in resistance and physiological effects will require further investigation. The observations in this study highlight the emergence and expansion of *vgsc* mutations and suggest the difficulty of vector control in Taiwan and worldwide. In addition, we found that mutations in Taiwan showed geographical differences.

## Introduction

*Aedes aegypti* is the primary vector of various arboviruses, including dengue, chikungunya, Zika, and yellow fever viruses. Of these, dengue virus causes a spectrum of diseases from asymptomatic dengue fever to severe dengue, including hemorrhagic fever and shock syndrome with high mortality [1]. Approximately 390 million people are estimated to be infected with dengue annually, causing health and economic burdens worldwide [2]. However, global transit and climate change have promoted the geographical expansion of both dengue vectors and dengue virus, increasing the global risk of arboviral disease [3,4]. In Taiwan, *Ae*. *aegypti* is limited to southern Taiwan, which is a hotspot of the dengue transmission [5]. According to surveillance by the Taiwan Centers for Disease Control (Taiwan CDC; https://www.cdc.gov.tw/), dengue has been detected annually over the past two decades. Record levels were reached in 2015, with 43,419 confirmed indigenous cases and more than 98.5% of cases occurring in *Ae*. *aegypti*-endemic areas, including Tainan, Kaohsiung, and Pingtung. However, dengue is considered a travel disease in Taiwan because most dengue outbreaks have emerged from cases imported in early summer, with subsequent virus spread by local vector populations [6]. Outbreaks usually end in winter when the weather becomes cold and unsuitable for mosquito activity [7]. Taiwan closed its borders during the COVID-19 pandemic, and no indigenous dengue cases were identified in 2021. However, after the lockdown was lifted, an outbreak of re-emergent dengue resulted in 26,423 indigenous cases, with the majority of these cases in Taiwan in 2023 comprising DEN-1 infections.

Because specific medications and cost-effective vaccines are unavailable for dengue [8], mosquito control remains a primary strategy to combat dengue virus infection. New approaches such as sterile insect technology and *Wolbachia*-based and gene-modification strategies have been assessed in various projects [9–11]. Although a reduction in both mosquito populations and dengue incidence has been demonstrated in several areas, large-scale implementation is needed to fully assess the advantages and disadvantages of these innovative strategies. In Taiwan, a *Wolbachia* (*w*AlbB)-transinfected *Ae*. *aegypti* strain, *w*AlbB-Tw, was established for lab-scale characterization and semi-field assessment [12]. However, further assessments of *w*AlbB-Tw and detailed information on the local population are needed for a comprehensive evaluation before large-scale release. Therefore, insecticide spraying is still needed to interrupt viral transmission when a suspected case of dengue is reported [13]. Pyrethroids, categorized as type I or type II based on the presence of cyan groups, are the most commonly used insecticides because of their high toxicity in pests and minimal harm to mammals [14]. However, prolonged use of insecticides with the same mode of action results in resistance and hampers the efforts of vector control programs [15]. Similar to other Asian countries, in Taiwan, mosquitoes with low sensitivity to broad-acting insecticides have been observed among adult and larval *Ae*. *aegypti* [16–19].

Point mutations leading to non-synonymous amino acid substitutions in the voltage-gated sodium channel (*vgsc*), the pyrethroid receptor, result in knockdown resistance and have been discovered in various insects with public health and agricultural importance [20,21]. In *Ae*. *aegypti*, several *vgsc* mutations with distinct geographical distributions have been observed [22]. In Asia, associations between resistance and *vgsc* substitutions, including L982W, S989P, A1007G, T1520I, V1016G, F1534C, and D1763Y (positions are numbered based on housefly *vgsc*; GenBank accession number: AAB47604), have been reported in *Ae*. *aegypti*. These mutations confer resistance either alone or in combination with other mutations [23–27]. Co-occurrence of multiple *vgsc* mutations, which usually results in greater resistance, has been reported in many Asian countries. Kasai et al. reported super-insecticide-resistant *Ae*. *aegypti* carrying substitutions L982W+F1534C that showed 300-fold resistance to permethrin. In addition, S989P+V1016G+F1534C was associated with approximately 170-fold greater resistance to permethrin and deltamethrin [27]. The Taiwan CDC launched a long-term surveillance program to monitor the resistance of field populations of *Ae*. *aegypti* in 2016, and four *vgsc* mutations (S989P, V1016G, F1534C, and D1763Y) and two intron polymorphisms (250 or 234 bp of intron inserts between exon 20 and 21 of *vgsc* domain II region) were detected [25,28]. By these four mutations and two intron polymorphisms, six haplotypes were proposed in *Ae*. *aegypti* in Taiwan. Among these, S989P+V1016G is associated with resistance to several type II pyrethroids [18,28]. In 2022, Chung et al. described six resistance-associated and several resistance-unrelated genotypes in *Ae*. *aegypti* in Taiwan [29]. Several mutations associated with resistance, including L982W, A1007G, and T1520I, have been reported in other countries in recent years; however, these mutations have yet to be investigated in Taiwan [24,26,27]. Therefore, this study aimed to monitor the current resistance status of *Ae*. *aegypti* in Taiwan. Long-term surveillance data were used to understand how *vgsc* mutations varied temporally and geographically under a sustained vector control program.

## Materials and methods

### Mosquito collection and maintenance

**Fig 1:**
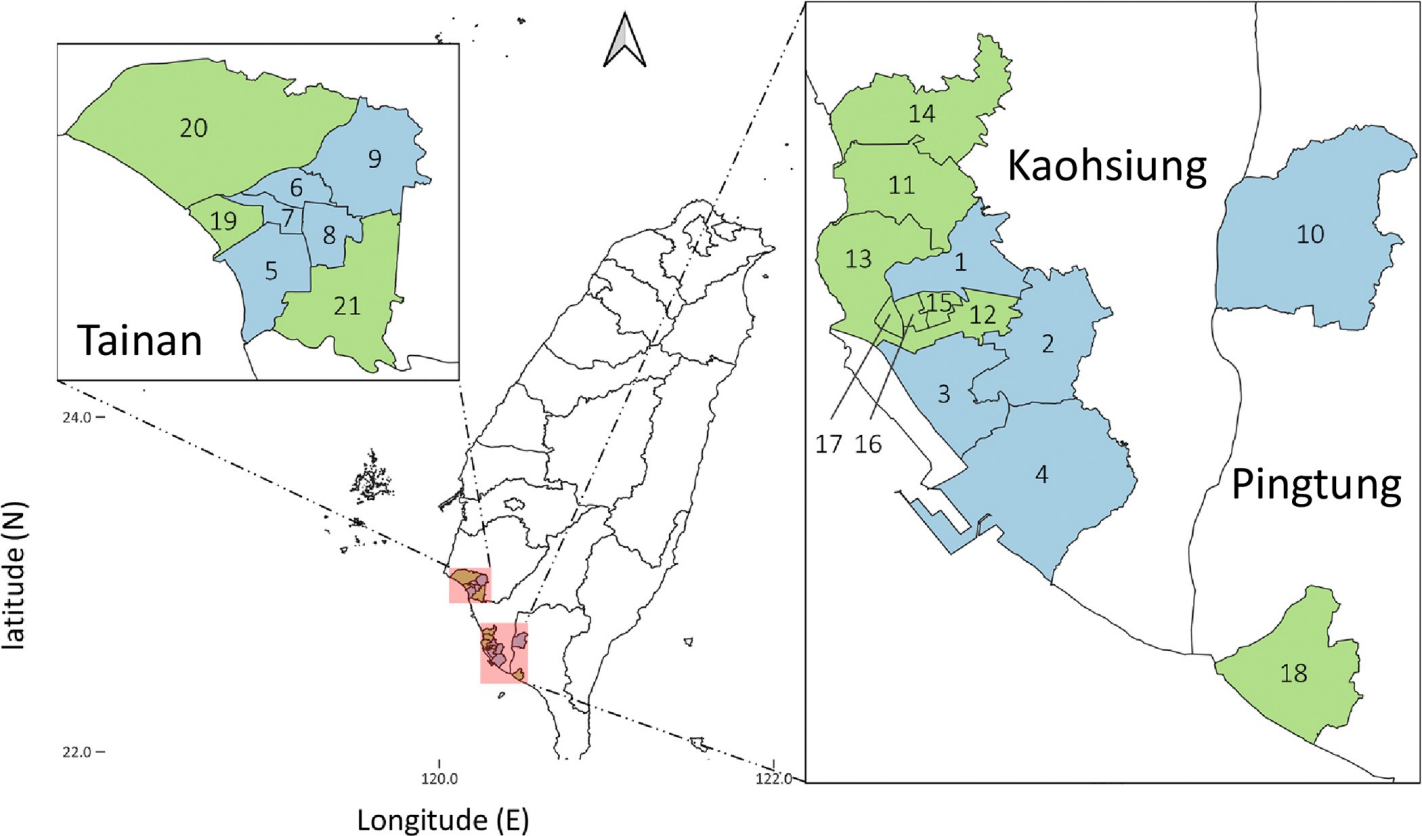
Map of *Aedes aegypti* sampling sites. Routine surveillance in 10 districts of Tainan City, Kaohsiung City, and Pingtung County in southern Taiwan that are labeled in blue. The expanded districts (those added in 2019) are shown in green. Sanmin (1), Fengshan (2), Qianzhen (3), Xiaogang (4), South (5), North (6), West Central (7), East (8), Yongkang (9), Pingtung (10), Zuoying (11), Lingya (12), Gushan (13), Nanzih (14), Hsingsin (15), Chiengin (16), Yancheng (17), Donggang (18), Anping (19), Annan (20), and Rende (21). The map was created in QGIS 3.32.2 (https://qgis.org). The base layer of the map with CC BY 4.0 license (https://data.gov.tw/licenses) was downloaded from Government Open Data established by National Development Council, Taiwan (https://data.gov.tw/dataset/7442).

Immature *Aedes* mosquitoes were collected from standing water containers or ovitraps set by the National Health Research Institute in March (2^nd^ season) and October (4^th^ season) between 2016 and 2023. However, the survey was not conducted in October 2019 due to a policy adjustment. These collections were conducted in 10 districts at high risk for dengue in southern Taiwan, including five districts of Tainan City (West Central District, South District, North District, East District, and Yongkang District), four districts of Kaohsiung City (Sanmin District, Xiaogang District, Qianzhen District, and Fengshan District), and Pingtung City in Pingtung County. Because of the disappearance of wild-type genotype in 2018, in 2019, we expanded sampling to 21 sites in Tainan (n = 8), Kaohsiung (n = 11), and Pingtung (n = 2) to confirm *vgsc* fixation (Fig 1). Mosquito species were identified under a dissecting microscope as described previously [30]. The mosquitoes were then reared to adulthood in an insectary following a previously reported procedure [28]. Briefly, larvae were reared in a plastic pan containing a 3:1 mixture of pig liver powder and yeast extract. Adult mosquitoes were kept in a BugDorm screen cage (30 × 30 × 30 cm; MegaView Science, Taichung, Taiwan) under conditions of a 10:14 light:dark cycle, 20–30°C, and 70 ± 10% relative humidity. A 10% sucrose solution was used as an energy source for adult mosquitoes. G0 males were selected, preserved in absolute ethanol, and stored at –80°C until *vgsc* genotyping.

### *vgsc* genotyping

To prevent sperm contamination in female spermatheca, genomic DNA was extracted from field G0 males using a Qiagen QIAamp DNA purification kit (cat. no. 51306, Qiagen, Germany). Because of the difference in the location of the sex determination factor (chromosome 1) and the *vgsc* gene (chromosome 3), theoretically, there should be no gender bias based on results from male mosquitoes [31]. Briefly, each mosquito was individually homogenized with a 3-mm glass bead in a 1.5-mL microcentrifuge tube for 3 min at a frequency of 30/s using a TissueLyser (Qiagen, Germany). The homogenized sample was then processed according to the manufacturer’s instructions, and genomic DNA was eluted with 80 μL Tris-EDTA buffer. The *vgsc* gene was genotyped as described previously [28,32]. At the beginning of 2016, partial DNA fragments of *vgsc* containing S989, V1016 (domain II), F1534 (domain III), and D1763Y (domain IV) were amplified using three sets of PCR primers (S1 Table) [32] and a thermocycler (Biometra T3000, Germany). PCR was conducted with 12.5 μL 2*×* PCR Master mix solution (i-pfu) (cat. no. 25186, iNtRON Biotechnology, Korea), 1 μL each forward and reverse primers (10 μM), 1 μL genomic DNA, and 9.5 μL ddH_2_O in a final volume of 25.0 μL. PCR conditions were as follows: 94°C for 5 min, followed by 39 cycles of denaturation at 94°C for 30 s, annealing at 55°C for 30 s, extension at 72°C for 1 min, and a final extension step at 72°C for 10 min. Specific PCR amplification products were separated using electrophoresis on a 1.5% agarose gel and visualized on an ultra-violet light box following ethidium bromide staining. Amplicons were sent for direct sequencing (Genomics, Taiwan) using the designated sequencing primers (S1 Table) [32]. The *vgsc* genotypes of the four alleles were aligned and analyzed using GeneStudio software (http://genestudio.com/). Haplotypes and genotypes were determined as described in previous reports [28,29]. Following the reported resistance roles of T1520I (2019), A1007G (2021), and L982W (2022), we searched for these mutations in previous results for domain II and III sequencing, and these mutations were included in subsequent testing. We genotyped 833, 749, and 179 male mosquitoes collected in Tainan, Kaohsiung, and Pingtung, respectively.

### Statistical analysis

All statistical analyses were performed using Prism version 6.01 (GraphPad Software Inc.). The differences in allele (S989P, V1016G, T1520I, F1534C, and D1763Y), haplotype, and genotype distributions in *Ae*. *aegypti* were compared between Tainan, Kaohsiung, and Pingtung. Analysis of variance (ANOVA) was employed, and *post-hoc* Tukey testing was used for multiple comparisons if significant differences were observed.

## Results

### *vgsc* mutations

Between 2016 and 2023, eight *vgsc* mutations (L982W, S989P, A1007G, I1011M, V1016G, T1520I, F1534C, and D1763Y) were monitored by genotyping of 1,761 field-caught male *Ae*. *aegypti* from southern Taiwan. Five of these eight mutations were detected: S989P, V1016G, T1520I, F1534C, and D1763Y. Of these mutations, V1016G exhibited the highest mutation frequency (average: 0.44, range: 0.16–0.59), followed by F1534C (0.42, 0.15–0.61), S989P (0.31, 0.06–0.51), D1763Y (0.14, 0.04–0.19), and T1520I (0.04, 0–0.13) (S2 Table). However, mutations in L982, A1007, and I1011 were not detected in *Ae*. *aegypti* from southern Taiwan.

**Fig 2:**
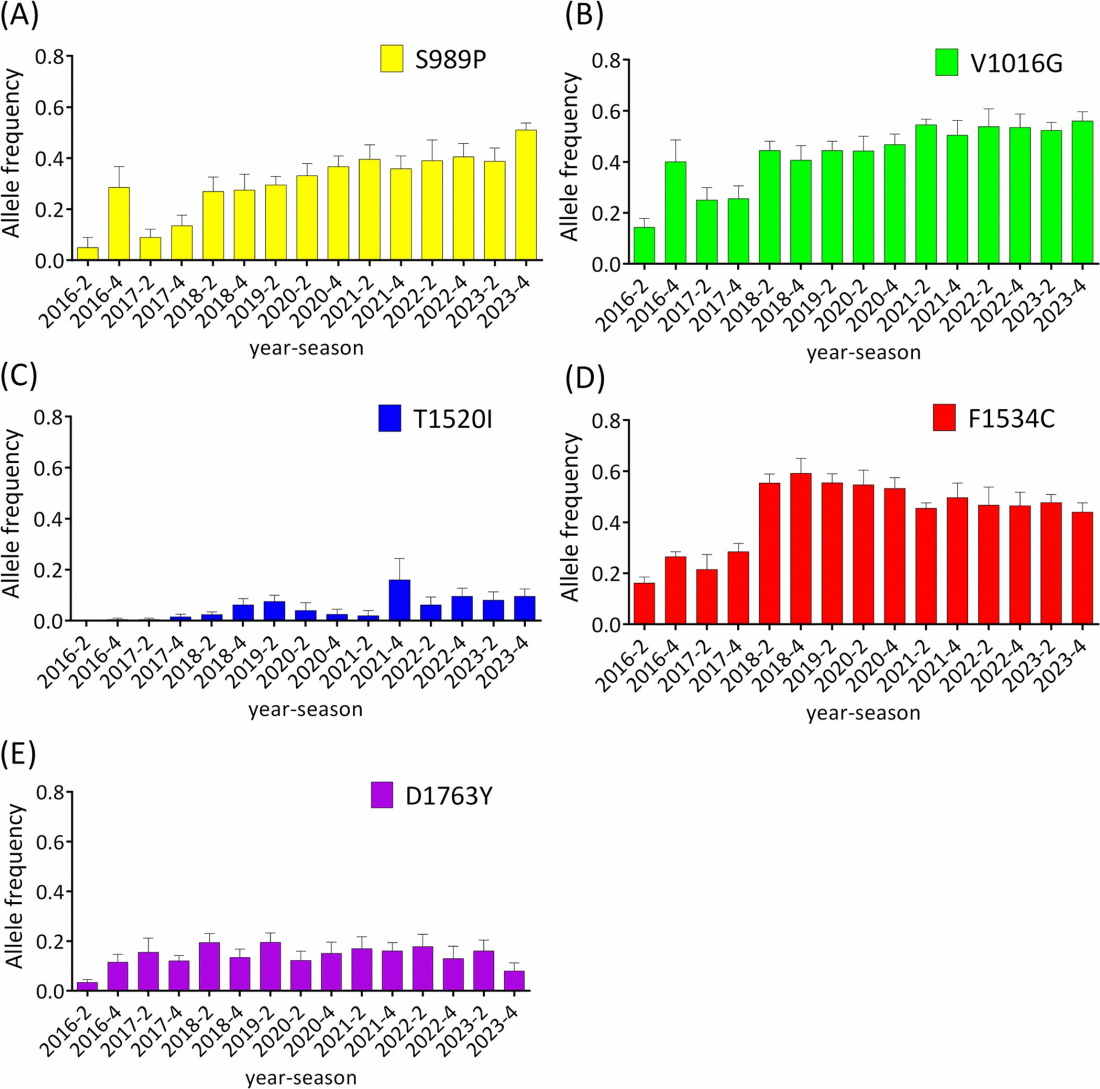
Temporal analysis of the frequencies of mutations in *Aedes aegypti*. The histogram displays the frequencies of S989P (A), V1016G (B), T1520I (C), F1534C (D), and D1763Y (E) mutations in *Ae*. *aegypti*. The means with standard errors of the mean are depicted for *Ae*. *aegypti* mutation frequencies in each district. The average allele frequencies of S989P, V1016G, T1520I, F1534C, and D1763Y in 2019 with the expanded areas used to plot this figure (0.26, 0.39, 0.05, 0.61, and 0.13) were similar to those without expanded areas (0.27, 0.40, 0.07, 0.61, and 0.14).

T1520I was initially detected at a low frequency (0.004 in October 2016); however, its frequency gradually increased thereafter (Fig 2C). The frequency of V1016G increased steadily since March 2017, and it has become the predominant mutation in recent years (Fig 2B). The frequency of S989P varied concurrently with that of V1016G but at a relatively lower frequency (Fig 2A). The frequency of F1534C peaked in October 2018, and the three-year trend of increase was reversed in subsequent years (Fig 2D). The frequency of D1763Y remained stable at approximately 0.15 throughout the period of surveillance (Fig 2E).

**Fig 3:**
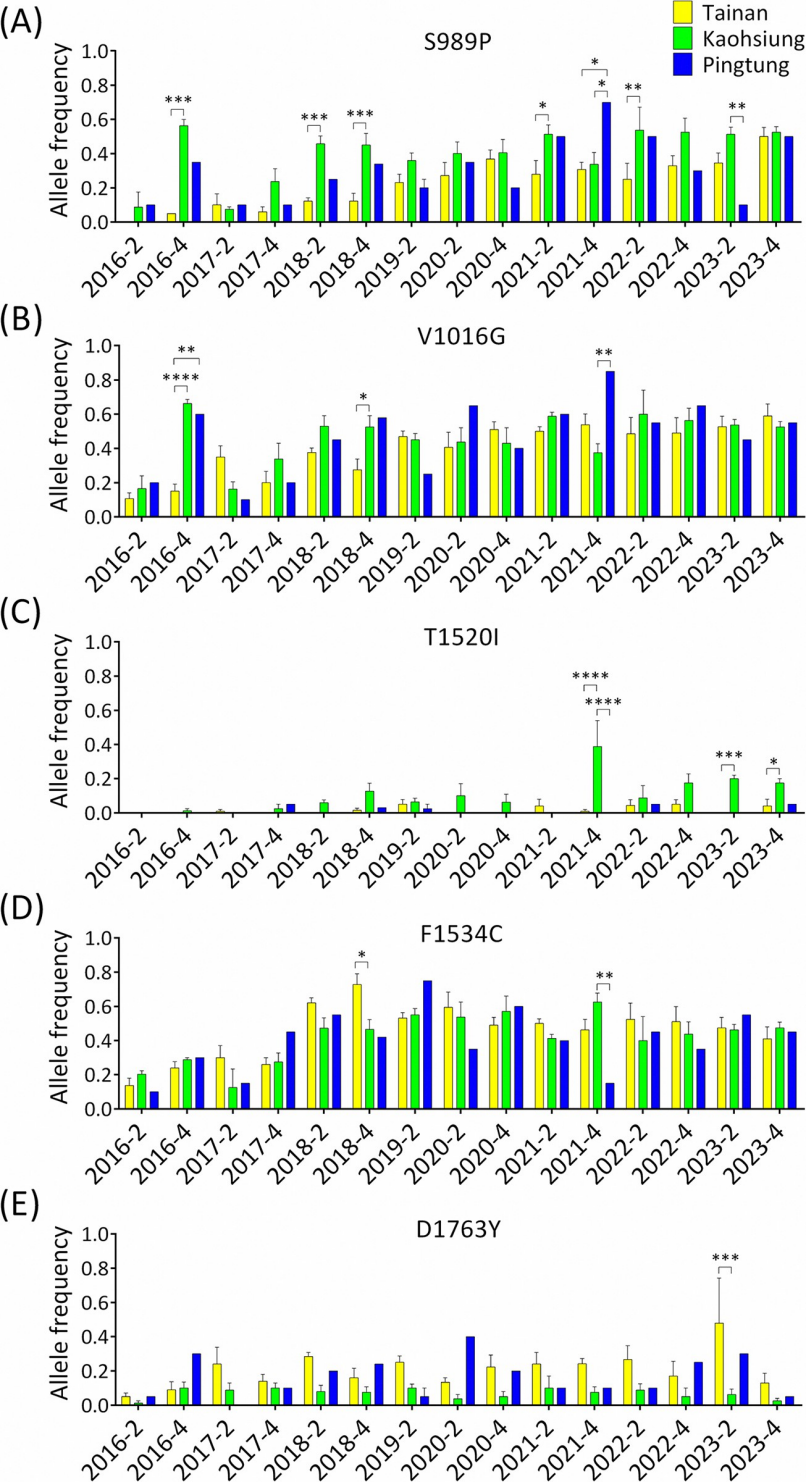
Temporal analysis of the mutation frequencies of S989P (A), V1016G (B), T1520I (C), F1534C (D), and D1763Y (E) in *Aedes aegypti* collected in Tainan, Kaohsiung, and Pingtung. The means with standard errors of the means were plotted in the histogram according to the frequencies of *Ae*. *aegypti* mutations in each district (* *p <* 0.05; ** *p <* 0.01; *** *p <* 0.005; **** *p <* 0.001).

Regarding the geographical distribution of these mutations, the frequency of S989P was generally higher in Kaohsiung than in Tainan or Pingtung, especially at particular time points where these differences were statistically significant (Fig 3A). The frequency of V1016G in Tainan was lower than that in Kaohsiung and Pingtung before 2018, with significant differences observed in October 2016 and 2018. However, there were no significant differences in V1016G frequencies among the three cities over the last two years of the study (Fig 3B). Notably, both S989P and V1016G were detected more frequently in Pingtung in October 2021 than in Tainan and Kaohsiung (Fig 3A and 3B). The frequency of T1520I was significantly higher in Kaohsiung than in Tainan and Pingtung, especially after October 2021 (Fig 3C). The frequencies of F1534C in the three cities were relatively similar, except in October 2018 and 2021 (Fig 3D). In contrast to the frequency of S989P and V1016G, the frequency of D1763Y was higher in Tainan than in Kaohsiung, especially in March 2023, and the difference was statistically significant (Fig 3E).

**Fig 4:**
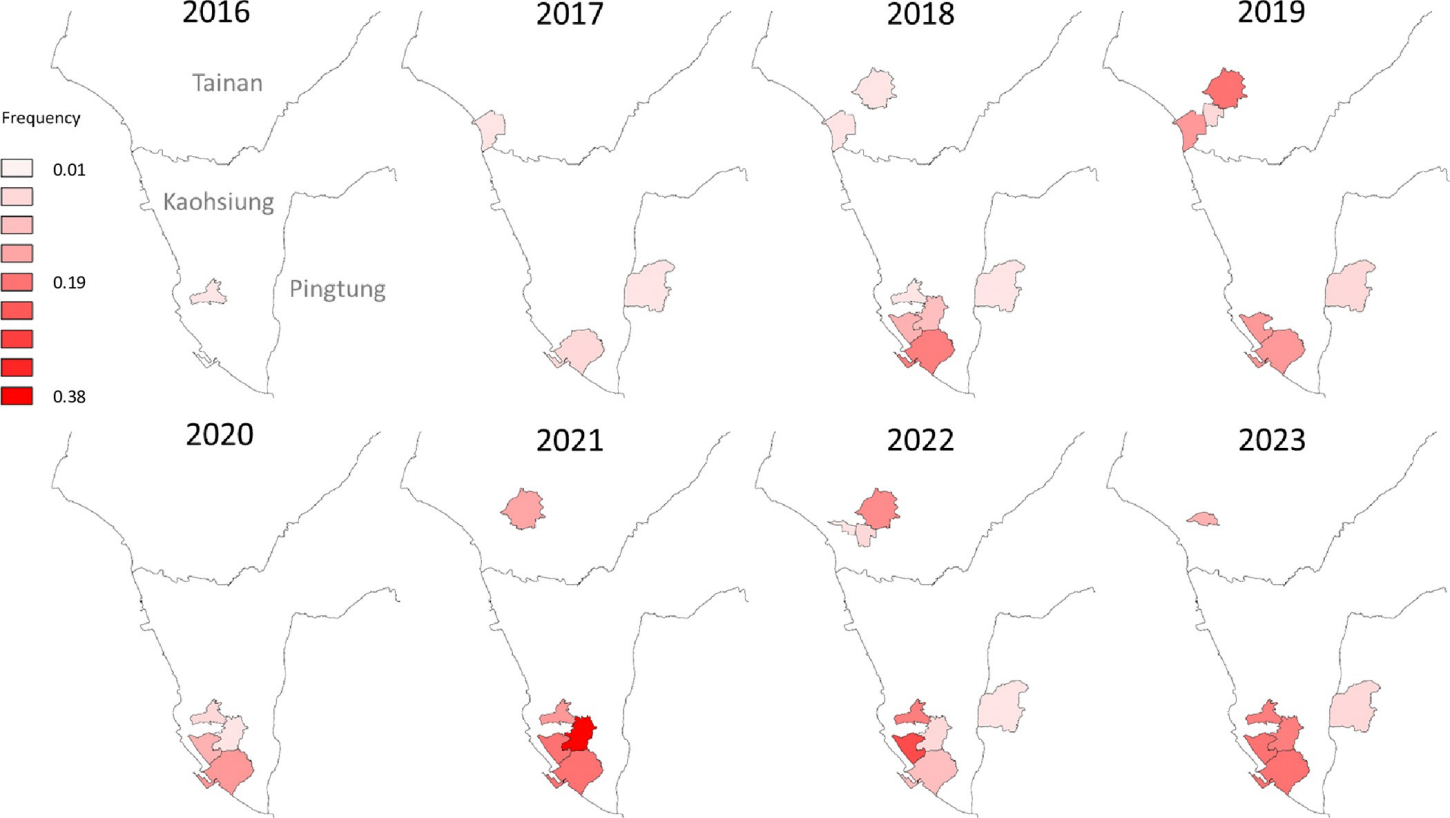
Temporal and spatial analysis of T1520I in *Aedes aegypti* in Taiwan. The expanded districts in 2019 were not included in this analysis. The map was created in QGIS 3.32.2 (https://qgis.org). The base layer of the map with CC BY 4.0 license (https://data.gov.tw/licenses) was downloaded from Government Open Data established by National Development Council, Taiwan (https://data.gov.tw/dataset/7442).

T1520I was first detected in *Ae*. *aegypti* in Taiwan in this study, and analyses of the spatial and temporal distribution of this mutation were conducted. This mutation was first detected in a district of Kaohsiung City (Sanmin) in 2016 at a low frequency of 0.002. The following year, T1520I spread to Tainan and Pingtung, and mosquitoes with T1520I were found in three districts. The average frequency increased by 8.3-fold to 0.017. Subsequently, this mutation rapidly spread to seven of the districts sampled in southern Taiwan in 2018. In the past two years, T1520I was detected in most of the districts monitored in this study, with average frequencies ranging from 0.07 to 0.08, representing a 35- to 40-fold increase after its emergence (Figs 2C and 4).

### *vgsc* genotypes

**Fig 5:**
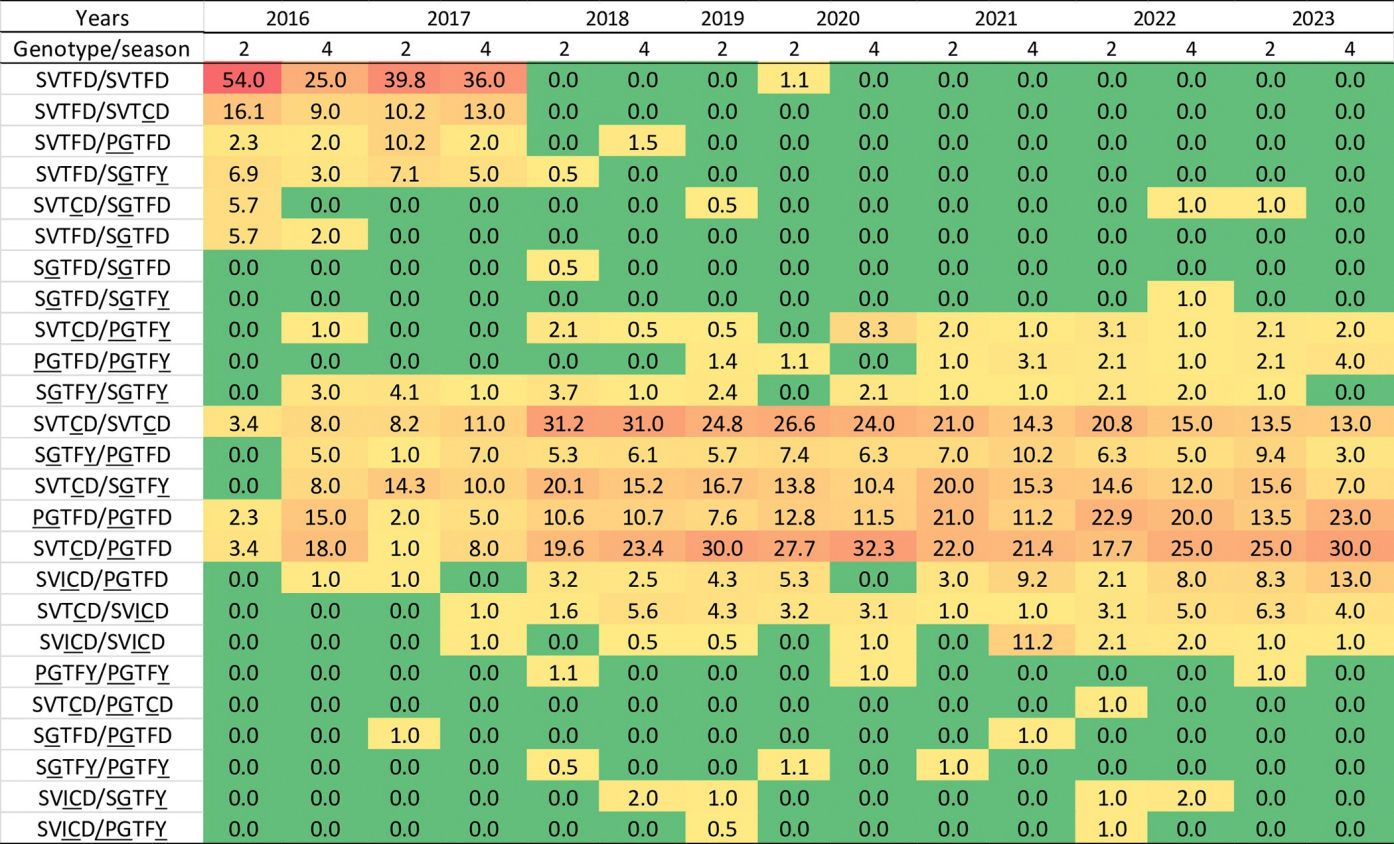
Heatmap of the distribution of *vgsc* genotypes in *Aedes aegypti* collected in Taiwan between 2016 and 2023. The proportion from low to high is presented by colors in the order of green < orange < red. Underlined letters represent the mutant alleles in each position.

The composition of *vgsc* genotypes reflects the ecological balance between fitness and resistance in mosquitoes exposed to insecticides. During this survey, 25 *vgsc* genotypes (unmutated wild-type mosquitoes with intron A or B polymorphisms were classified as the same genotype in this study), comprising S989P, V1016G, T1520I, F1534C, and D1763Y, were observed in natural populations of *Ae*. *aegypti* (Fig 5). Among these, 14 genotypes had been previously reported in Taiwan; the presence of the remaining genotypes was documented for the first time in Taiwan in this study. Notably, at the beginning of this surveillance study, the most frequent genotype was the unmutated wild type (SVTFD/SVTFD), which was detected in 54% of the field mosquitoes. However, this genotype disappeared in 2018, despite our increase in sampling that year and the inclusion of additional districts in 2019 for confirmation of this disappearance (with only 1.1% wild-type field-collected mosquitoes observed in March 2020). Simultaneously, the population with previously reported [29] resistance-unrelated genotypes (SVTFD/SVTCD, SVTFD/PGTFD, SVTFD/SGTFY, SVTCD/SGTFD, and SVTFD/SGTFD) decreased to undetectable levels over time. In contrast, the number of *Ae*. *aegypti* with one of the five resistance-related *vgsc* genotypes (SVTCD/SVTCD, SGTFY/PGTFD, SVTCD/SGTFY, PGTFD/PGTFD, and SVTCD/PGTFD) described previously [29] increased to 76% of the field population in Taiwan. The SVTCD/PGTFD triple heterozygote population increased from 3.4% in March 2016 to 30% in October 2023, representing an 8.8-fold increase, to become the predominant genotype in the field. Similarly, the frequency of PGTFD/PGTFD homozygote increased by ten-fold (2.3% to 23%). The SVTCD/SVTCD homozygote was first detected at a frequency of 3.4%, and the population rapidly increased to a peak of 31.2% in March 2018. The prevalence of this genotype gradually decreased to 13% by 2023. With trend similar to SVTCD/SVTCD but at a relatively low frequency, the SGTFY/PGTFD population peaked in October 2021 and then decreased over time. The frequency of SVTCD/SGTFY ranged from 7% to 20.1%, with peaks observed in March 2018 and March 2021. In addition, the SVICD/PGTFD population rapidly expanded (13-fold) after its first detection in 2016, suggesting its role in resistance and conferring a survival advantage. We also detected a slight increase in the SVTCD/SVICD population (approximately 5-fold). In contrast, three genotypes, PGTFY/PGTFD, SGTFY/SGTFY, and SVICD/SVICD, which were absent at the beginning of our surveillance, emerged later and were maintained at a low frequency. Other genotypes were detected occasionally, including the PGTFY/PGTFY triple-homozygous mutation, which was first reported in *Ae*. *aegypti* in this study.

### *vgsc* haplotypes

**Fig 6:**
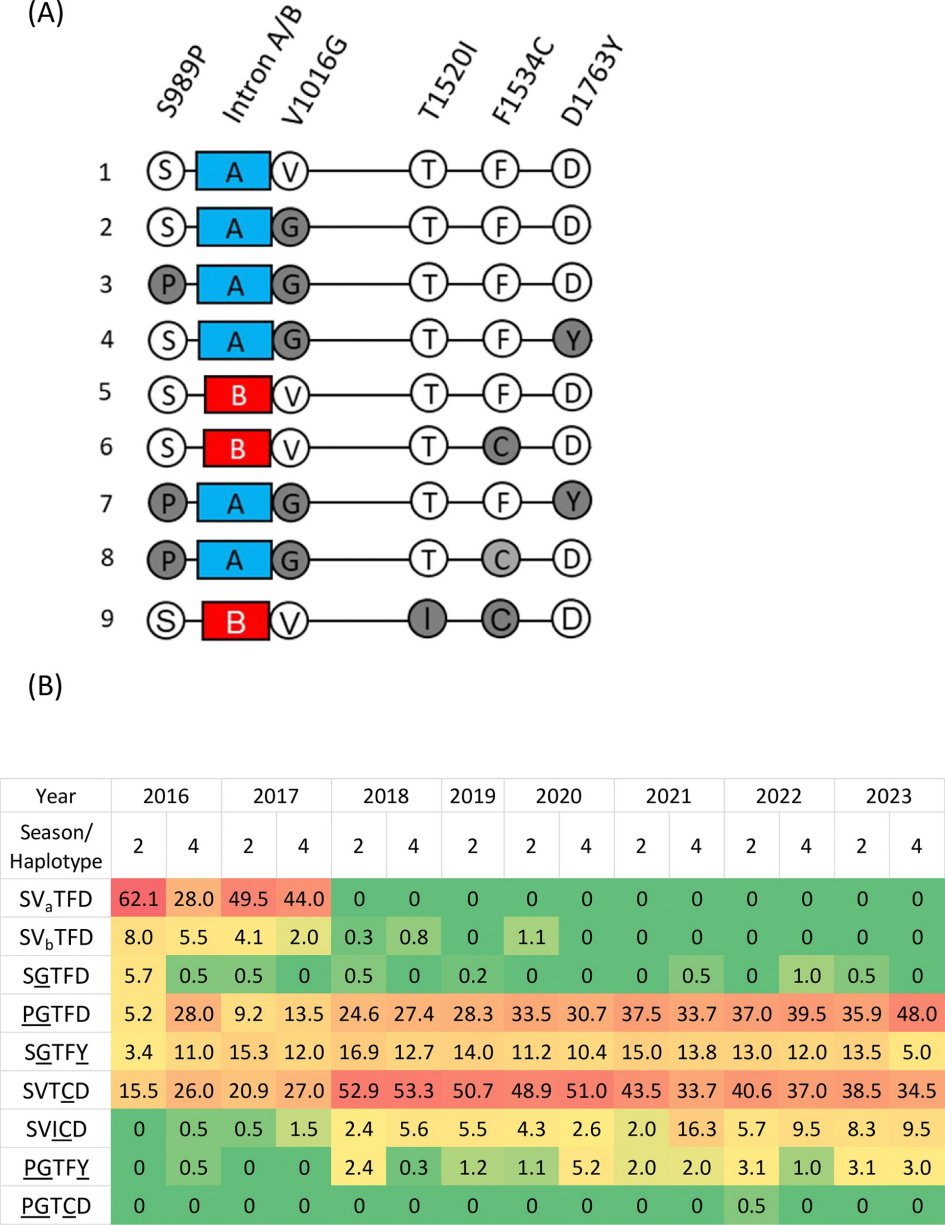
**Proposed haplotypes (A) and heatmap of the distribution of haplotypes (B) in *Aedes aegypti* collected in Taiwan between 2016 and 2023.** Wild-type and mutation sites are presented in white and gray circles, respectively. Blue and red boxes represent group A and B introns, respectively. a and b denote two types of intron polymorphisms. The underlined letter represents the mutant alleles in each position.

**Fig 7:**
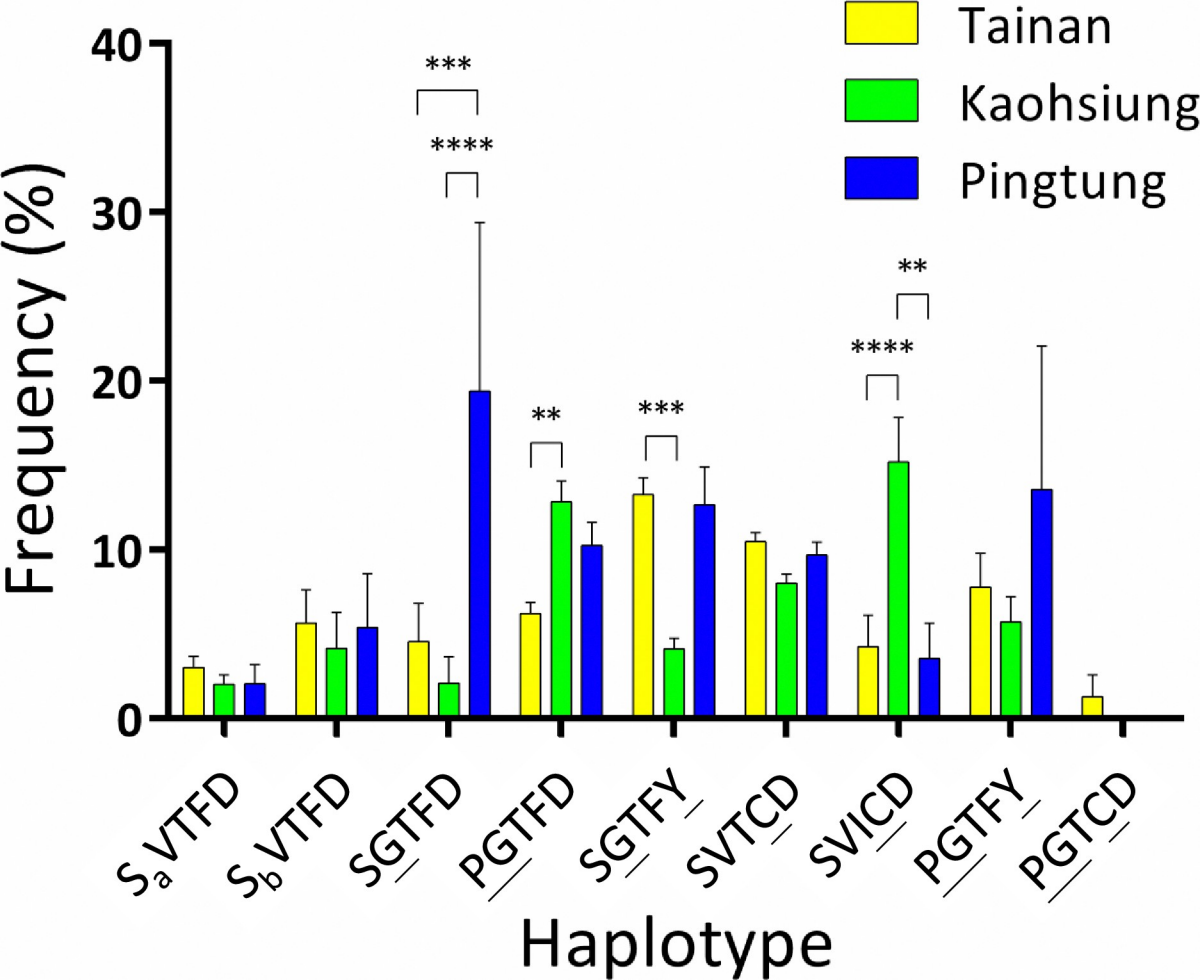
Distribution of *Aedes aegypti* haplotypes in Tainan, Kaohsiung, and Pingtung. The means and standard errors of the means were plotted according to the frequencies of *Ae*. *aegypti* haplotypes in each district. (** *p <* 0.01; *** *p <* 0.005; **** *p <* 0.001). The underlined letter represents the mutant allele in each position. a and b denote two types of intron polymorphisms.

Based on genotyping, we identified nine *Ae*. *aegypti* haplotypes with five mutations: S989P, V1016G, T1520I, F1534C, and D1763Y (Fig 6A). In the time-course analysis (Fig 6B), the unmutated haplotype (SVTFD) accounted for the vast majority (70%) of the field population at the beginning of the surveillance. However, this haplotype had completely disappeared by 2020. The SGTFD haplotype was first detected at a frequency of 5.7% in March 2016; however, it was rarely detected (<1%) during subsequent surveillance. In contrast, the frequency of PGTFD increased by 10.5-fold, accounting for 48% of the *Ae*. *aegypti* collected. SVTCD frequency peaked at 53% in 2018 and then gradually decreased to 34.5% by 2023. These two haplotypes constituted 82.5% of the field population in 2023. SGTFY was continuously observed in the field and was particularly prevalent between October 2016 and March 2023, with frequencies ranging from 10.4% to 16.9%. In addition, three haplotypes, PGTFY, SVICD, and PGTCD, were detected for the first time in Taiwan. PGTFY was first detected in October 2016, and its frequency peaked at 5.2% in October 2020. This haplotype was then maintained at a low frequency (1–3%) in the following years. SVICD was first detected in October 2016 at a low frequency of 0.5%. However, this haplotype continually expanded, reaching 9.5% (18.6-fold) as the SVTCD frequency began to decline. We only detected PGTCD in a single individual in 2022. Additionally, we analyzed the distribution of the nine observed haplotypes among the three cities (Fig 7). SGTFD mainly existed in Pingtung. The frequency of SGTFY in Tainan was significantly higher than that in Kaohsiung, while the frequency of PGTFD in Kaohsiung was considerably higher than that in Tainan. The newly emerging haplotype SVICD was predominant in Kaohsiung. Differences in the other haplotypes were not significantly different among the three cities.

### *vgsc* genotype distribution

**Fig 8:**
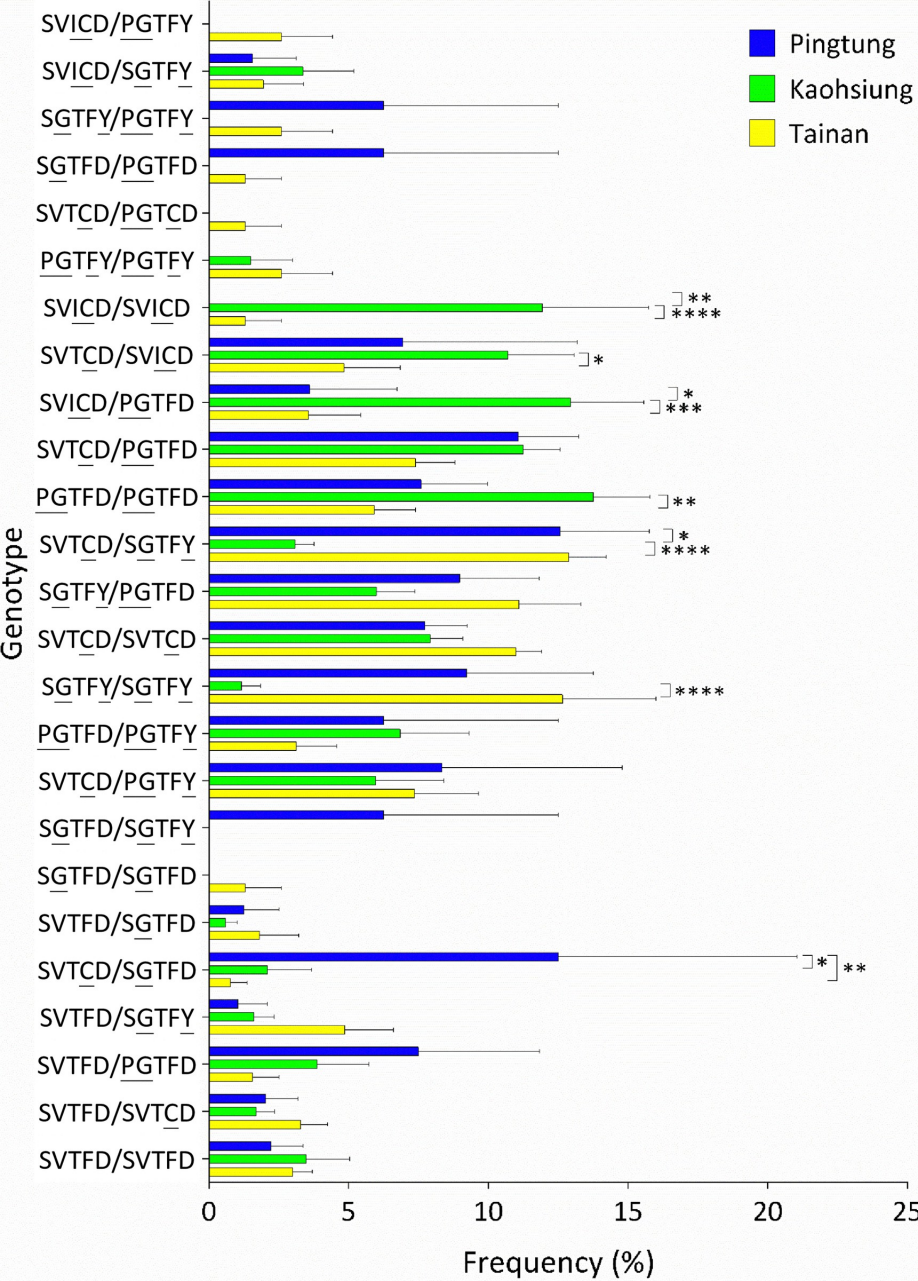
Distribution of *Aedes aegypti* genotypes among Tainan, Kaohsiung, and Pingtung. The means and standard errors of the means were plotted according to the frequencies of *Ae*. *aegypti* genotypes in each district. (* *p <* 0.05; ** *p <* 0.01; *** *p <* 0.005; **** *p <* 0.001). Underlined letters represent the mutant alleles in each position.

When we analyzed the distribution of genotypes among the three cities, it was not surprising that genotypes comprising the haplotypes SVICD, PGTFD, and SVTCD were significantly more frequent in Kaohsiung than in Tainan and/or Pingtung. Frequencies of SGTFY/SGTFY in Tainan and SVTCD/SGTFY in Tainan and Pingtung were significantly higher than those in Kaohsiung. The SGTFD/SVTCD frequency was significantly higher in Pingtung than in Tainan and Kaohsiung (Fig 8).

## Discussion

In this study, surveillance of insecticide resistance was conducted based on the detection of *vgsc* mutations in *Ae*. *aegypti* in Taiwan from 2016 to 2023. The substitutions S989P, V1016G, T1520I, F1534C, and D1763Y were detected in the field population. Among these, T1520I was identified for the first time in *Ae*. *aegypti*, and its frequency and distribution expanded across southern Taiwan over the course of surveillance. The frequencies of the other four mutations also increased over time. The most prevalent mutation was V1016G, followed by F1534C. Five resistance-associated genotypes, SVTCD/SVTCD, SGTFY/PGTFD, SVTCD/SGTFY, PGTFD/PGTFD, and SVTCD/PGTFD, were found to represent the vast majority of *Ae*. *aegypti* in the field. Additionally, we observed an emerging genotype, SVICD/PGTFD, and its frequency increased 13-fold over the surveillance period. Conversely, the unmutated haplotype disappeared completely after 2020. This suggests that *vgsc* mutations, at least one in each individual, became fixed in *Ae*. *aegypti* in Taiwan. The dominant haplotypes were PGTFD (48%), followed by SVTCD (34.5%), and SVICD (9.5%), with these three haplotypes accounting for 92% of the population. These trends indicated decreasing haplotype diversity in the field population. In this study, we detected PGTFY for the first time in *Ae*. *aegypti*. Moreover, we noted geographical differences in mutations, suggesting the need to adjust vector control strategies based on local resistance data.

Mutations in *vgsc* associated with pyrethroid resistance have been widely reported in *Ae*. *aegypti*. Previous studies have reported that these mutations confer pyrethroid resistance, either independently or in combination with other mutations [23,26,27,33,34]. In Taiwan, four mutations (S989P, V1016G, F1534C, and D1763Y) have been documented in field populations of *Ae*. *aegypti* [28]. During our surveillance, we observed an increase in the frequency of these four mutations over time, indicating a shift toward higher levels of resistance in the field population. Furthermore, we identified another mutation, T1520I, for the first time in *Ae*. *aegypti* in Taiwan. This mutation was initially detected in *Ae*. *aegypti* in India in 2015 [35] and later spread to neighboring countries, including Laos, Myanmar, and Pakistan. According to previous studies, T1520I typically occurs in combination with F1534C [36–39]. Functional bioassay indicated that T1520I enhances F1534C-mediated insensitivity [26]. Consistent with these findings, T1520I always occurred in the presence of F1534C in our study. In addition, T1520I increased in frequency (13-fold) over the study period, expanding its distribution in Taiwan. These observations suggest that the resistance-associated mutation T1520I emerged and was amplified within *Ae*. *aegypti* populations on isolated islands of Taiwan. This finding should be considered when assessing the resistance status of *Ae*. *aegypti*. However, further investigation is needed to determine whether this mutation was imported with foreign mosquitoes or originated as a *de novo* mutation in local populations and whether T1520I can enhance the fitness of mosquitoes with F1534C. On the other hand, a previous study reported that D1763Y co-occurs with V1016G to confer resistance [40]. However, our surveillance showed that D1763Y retained a relatively low frequency compared to S989P, V1016G, and F1534C.Therefore, the detailed impact of D1763Y on the physiological effects and resistance needs further elucidation.

In a previous study, six haplotypes, consisting of four mutations and two intron polymorphisms, were identified in *Ae*. *aegypti* in Taiwan. Other studies have demonstrated the association between the PGTFD haplotype and pyrethroid resistance [18,28]. In this study, we propose three new haplotypes that are present in Taiwan. The first is a haplotype harboring T1520I and F1534C, which exhibited a 19-fold increase after its first detection in 2016. This haplotype co-circulated with the resistance-associated haplotype PGFTD in field mosquitoes, suggesting a resistance role for T1520I+F1534C, which is consistent with previous findings [26]. The second newly proposed haplotype is PGTFY, which was observed in *Ae*. *aegypti* for the first time in this study. In previous studies, both PG and GY haplotypes were found to be associated with resistance to *Ae*. *aegypti* in Taiwan and in other countries [25,28,41]. Existing evidence indicates that the co-occurrence of multiple mutations enhances resistance. For example, PGC triple mutation shows 11- and 44-fold greater resistance to permethrin than those with PG or C mutations in *Xenopus* oocytes expression system [41]. However, previous studies have suggested that multiple mutations may confer a fitness cost in *Ae*. *aegypti*, which can limit the expansion of unfavorable mutations [42]. In this study, the PGTFY haplotype was maintained at low frequencies, ranging from 0.25% to 5.21%, over the five years of surveillance. Although PGTFY co-circulated with other resistance-associated haplotypes, its low frequency implied that *Ae*. *aegypti* with this haplotype may not be highly suitable for environmental expansion. To our knowledge, this is the first report documenting this haplotype in *Ae*. *aegypti*. Further investigations are needed to assess the physiological and resistance effects of PGTFY. In addition, the PGTCD haplotype was first detected in *Ae*. *aegypti* in Taiwan in combination with SVTCD. Fortunately, this haplotype was not detected in subsequent years, suggesting that mosquitoes harboring PGTCD did not expand in the field. This result aligns with those of previous studies, which indicated that PGC leads to a fitness cost in *Ae*. *aegypti* and is not advantageous for survival in the field. However, a single crossing-over event between the haplotypes harboring PG and C can lead to the formation of the PGC haplotype [41]. We observed that PGTFD, SVICD, and SVTCD accounted for 92% of the field population in 2023, compared with 20.7% at the beginning of surveillance. This observation indicates the increasing likelihood of the re-emergence of this super-resistant haplotype. In addition, PGTFD, SVTCD, and SGTFY have been reported to be associated with resistance [25,28,43]. In our surveillance, the haplotype SGTFY and its associated genotype constantly exhibited relatively low frequencies or showed a slight decline. We speculate that SGTFY would confer less resistance (or less fitness) than the PGTFD and SVTCD. However, the detailed role of SGTFY needs further investigation.

In this study, the frequencies of S989P, V1016G, and T1520I, as well as of the two haplotypes PGTFD and SVICD were significantly higher in Kaohsiung than in the other cities. The associations of these mutations with pyrethroid resistance have been demonstrated in previous studies [23,26,28]. Our results are consistent with those of a recent study, in which *Ae*. *aegypti* in Kaohsiung exhibited a notably higher insensitivity to pyrethroids than *Ae*. *aegypti* in Tainan [18]. Conversely, the D1763Y and SGTFY haplotype were predominant in the Tainan population. These observations were consistent with those of Lin et al., who reported that SGTFY-carrying *Ae*. *aegypti* mosquitoes were detected only in Tainan among the populations surveyed in southern Taiwan [18]. In October 2021, in Pingtung, significantly high frequencies of S989P and V1016G were accompanied by a decreased frequency of F1534C. It is reasonable to believe that F1534C and the former two mutations exist as different haplotypes, implying that selection pressure at that time shifted the population toward potently resistant P/G mutations. The opposite was also observed in Tainan in October 2018, implying that the population had shifted toward lower resistance [28]. In Taiwan, each city or county has its own local government, which formulates strategies for vector control according to local conditions. Factors influencing the geographical variation of *vgsc* mutations may include the historical pattern of insecticide usage, the temporal and spatial distribution of *Aedes*-borne disease, and the scale of the outbreak [18]. The distinct *vgsc* mutation pattern between cities is also consistent with previous studies [44]. Taken together, these results indicate that *vgsc* mutations in Taiwan are dynamic temporally and geographically. Therefore, it is essential to adjust the vector control strategy according to recent resistance information for local *Ae*. *aegypti*. However, the frequencies of F1534C and SVTCD were similar across Tainan, Kaohsiung, and Pingtung. This result is consistent with the understanding that F1534C is the most widespread resistance mutation in *Ae*. *aegypti* globally, and no obvious fitness cost for this mutation has been observed in previous studies [45,46].

The fact that unmutated wild-type *Ae*. *aegypti* disappeared in 2018 is intriguing, especially considering that there were only 13 indigenous dengue cases in that year compared to 43,419 and 15,492 cases in 2015 and 2014, respectively. We speculated that the absence of the unmutated genotype is partially attributable to the prophylactic chemical-based vector control that was conducted. To monitor the mosquito density effectively and prevent the occurrence of dengue outbreak, new vector control campaigns, including active mosquito surveillance using ovitraps, were introduced by local government authorities after 2016. Ovitraps were strategically set in a village, and the positive rate (ovitraps containing eggs of *Aedes* mosquito/total number of ovitraps) and the total number of *Aedes* eggs in each village were calculated weekly to guide environmental management decisions. If the positive rate exceeded 60% or the total number of *Aedes* eggs surpassed 500 in two consecutive weeks, a prophylactic chemical-based vector control strategy was considered to suppress the field population. Strategies aimed at maintaining mosquito populations at lower densities are beneficial for disease control. The numbers of indigenous dengue cases in southern Taiwan in 2016 (excluding the overwintering cases of the 2015 outbreak) and 2017 were 9 and 3, respectively. However, prophylactic insecticide spraying imposes selection pressures, accelerating the decline of unmutated wild-type mosquitoes in the field.

We have not been able to collect the unmutated wild-type *Ae*. *aegypti* since 2021. We also observed a shift toward higher frequencies of resistance-associated *vgsc* mutations. Previous studies have observed elevated fitness cost in *vgsc* mutant *Ae*. *aegypti* [47–49]. Studies also displayed the regain of the susceptibility and a decline of *vgsc* mutant frequency in the absence of insecticide exposure under laboratory conditions [47,50]. Whether the reversibility of resistance occurred in field *Ae*. *aegypti* in Taiwan needs long-term monitoring for clarification in the future. However, the expansion of resistance-associated mutations is an obstacle to the vector control program. Continued monitoring of the trend of *vgsc* mutations and evaluation of the insecticide efficacy during chemical-based intervention would be beneficial for effective vector control. We also suggest the authorities implement integrated pest management and insecticide resistance management to decelerate the evolution of resistance [51].

The relationship between insecticide resistance and vector competence of mosquitoes has also been investigated. *Aedes aegypti* with high frequencies of *vgsc* mutations V1016I and F1534C exhibited a longer survival time and an increased rate of dengue virus 1 dissemination [52]. Additionally, altered infection and dissemination rates were observed in Zika virus-infected *Ae*. *aegypti* carrying V1016I and F1534C mutations [53]. Increased transmission efficiency of the West Nile virus in *Culex quinquefasciatus* with either *ace-1* mutations or overproduction of carboxylesterase has been observed [54]. In the present study, there was a significant shift in the major genotype of field *Ae*. *Aegypti* from a specific unmutated wild-type to *vgsc*-mutated genotype. The PGTFD/SVTCD (30%) and PGTFD/PGTFD (23%) genotypes now constitute more than half the field population. Because of the unclear impact of these *vgsc*-mutated genotypes on *Ae*. *aegypti*, it is imperative to evaluate the association between these genotypes and vector competence to develop more effective disease prevention and control measures.

Studies have shown that *Wolbachia* introductions can benefit both vector control and disease prevention through both suppression and replacement of *Ae*. *aegypti* populations [55–57]. Berticat et al. reported that the *Wolbachia* density is higher in mosquitoes with certain resistance genomes, possibly because of the fitness cost of resistance mutations [58]. Furthermore, assessing the resistance status of local *Aedes* populations is a crucial prerequisite for releasing *Wolbachia*-carrying mosquitoes into the field [59,60]. In Taiwan, a *Wolbachia*-based biocontrol method was evaluated, and a *w*AlbB-transinfected *Ae*. *aegypti* strain, *w*AlbB-Tw, was established for lab-scale characterization and semi-field assessment [12]. In this study, we provide current resistance information on natural populations of *Ae*. *aegypti* in Taiwan, which is valuable for comprehensive evaluations in preparation for potential large-scale releases of *Wolbachia*-carrying *Ae*. *aegypti* to prevent arboviral diseases.

In conclusion, insecticide resistance remains a significant hurdle in chemical-based mosquito control programs. In this long-term surveillance study, a point mutation (T1520I) and three haplotypes, PGTFY (novel haplotype), SVICD, and PGTCD, associated with resistance were identified in *Ae*. *aegypti* in Taiwan. Over the study period, mosquitoes carrying resistance mutations expanded their territory and became fixed in *Ae*. *aegypti* in Taiwan. These findings indicate the widespread dissemination of resistance, highlighting the challenges to mosquito control in Taiwan and globally. In addition, our study revealed the dynamic evolution of *vgsc* resistance mutations, providing valuable information for monitoring resistance in areas where pyrethroids are used to control *Ae*. *aegypti*.

## Supporting information

S1 Table The PCR and sequence primers used in this study. (DOCX)

S2 Table Mutation frequencies of S989P, V1016G, T1520I, F1534C, and D1763Y of *Aedes aegypti* from Taiwan between 2016 and 2023. (XLSX)
