# Supplementary material for: First detection of the S989P+V1016G+D1763Y haplotype and expansion of voltage-gated sodium channel mutations in Aedes aegypti in Taiwan in 2016–2023
Source: PLoS Negl Trop Dis. 2025 Jan 6;19(1):e0012768. doi: 10.1371/journal.pntd.0012768 (PMC11737850; doi:10.1371/journal.pntd.0012768)
Supplement: S1 Table — (DOCX) [file pntd.0012768.s001.docx]

**S1 Table . The PCR and sequence primers used in this study**

| Primer Name | Sequence | Mutation sites |
| --- | --- | --- |
| AaSCF20(F) | GACAATGTGGATCGCTTCCC | L982W, S989P, A1007G, V1016G (PCR) |
| AaSCR21(R) | GCAATCTGGCTTGTTAACTTG |  |
| AaSCF3*(F) | GTGGAACTTCACCGACTTCA | L982W, S989P, A1007G, V1016G (Sequencing) |
| AaSCR22*(R) | TTCACGAACTTGAGCGCGTTG |  |
| AaSCF7(F) | GAGAACTCGCCGATGAACTT | T1520I, F1534C (PCR) |
| AaSCR7(R) | GACGACGAAATCGAACAGGT |  |
| AaSCR8(R) | TAGCTTTCAGCGGCTTCTTC | T1520I, F1534C (Sequencing) |
| AlSCF6(F) | TCGAGAAGTACTTCGTGTCG | D1763Y (PCR) |
| AlSCR8(R) | AACAGCAGGATCATGCTCTG |  |
| AlSCF7(F) | AGGTATCCGAACGTTGCTGT | D1763Y (Sequencing) |

*Both the AaSCF3 and AaSCR22 primers were used for domain II sequencing in case heterozygous intron polymorphisms existed between exons 20 and 21.
